# A machine learning tool to improve prediction of mediastinal lymph node metastases in non-small cell lung cancer using routinely obtainable [^18^F]FDG-PET/CT parameters

**DOI:** 10.1007/s00259-023-06145-z

**Published:** 2023-02-23

**Authors:** Julian M. M. Rogasch, Liza Michaels, Georg L. Baumgärtner, Nikolaj Frost, Jens-Carsten Rückert, Jens Neudecker, Sebastian Ochsenreither, Manuela Gerhold, Bernd Schmidt, Paul Schneider, Holger Amthauer, Christian Furth, Tobias Penzkofer

**Affiliations:** 1 Department of Nuclear Medicine, Charité – Universitätsmedizin Berlin, corporate member of Freie Universität Berlin and Humboldt-Universität Zu Berlin, Augustenburger Platz 1, 13353 Berlin, Germany; 2 Berlin Institute of Health at Charité – Universitätsmedizin Berlin, Berlin, Germany; 3 Department of Radiology, Charité – Universitätsmedizin Berlin, corporate member of Freie Universität Berlin and Humboldt-Universität Zu Berlin, Berlin, Germany; 4 Department of Infectious Diseases and Pulmonary Medicine, Charité – Universitätsmedizin Berlin, corporate member of Freie Universität Berlin and Humboldt-Universität Zu Berlin, Berlin, Germany; 5 Department of General, Visceral, Vascular and Thoracic Surgery, Charité – Universitätsmedizin Berlin, corporate member of Freie Universität Berlin and Humboldt-Universität Zu Berlin, Berlin, Germany; 6 Department of Hematology and Medical Oncology, Charité – Universitätsmedizin Berlin, corporate member of Freie Universität Berlin and Humboldt-Universität Zu Berlin, Berlin, Germany; 7 Charité – Universitätsmedizin Berlin, corporate member of Freie Universität Berlin and Humboldt-Universität Zu Berlin, Charité Comprehensive Cancer Center, Berlin, Germany; 8 Institute of Pathology, Charité – Universitätsmedizin Berlin, corporate member of Freie Universität Berlin and Humboldt-Universität Zu Berlin, Berlin, Germany; 9 Department of Internal Medicine – Pneumology and Sleep Medicine, DRK Kliniken Berlin Mitte, Berlin, Germany; 10 Department of Thoracic Surgery, DRK Kliniken Berlin Mitte, Berlin, Germany

**Keywords:** Lung cancer, NSCLC, Machine learning, Lymph node staging, FDG-PET/CT, Artificial intelligence

## Abstract

**Background:**

In patients with non-small cell lung cancer (NSCLC), accuracy of [^18^F]FDG-PET/CT for pretherapeutic lymph node (LN) staging is limited by false positive findings. Our aim was to evaluate machine learning with routinely obtainable variables to improve accuracy over standard visual image assessment.

**Methods:**

Monocentric retrospective analysis of pretherapeutic [^18^F]FDG-PET/CT in 491 consecutive patients with NSCLC using an analog PET/CT scanner (training + test cohort, *n* = 385) or digital scanner (validation, *n* = 106). Forty clinical variables, tumor characteristics, and image variables (e.g., primary tumor and LN SUVmax and size) were collected. Different combinations of machine learning methods for feature selection and classification of N0/1 vs. N2/3 disease were compared. Ten-fold nested cross-validation was used to derive the mean area under the ROC curve of the ten test folds (“test AUC”) and AUC in the validation cohort. Reference standard was the final N stage from interdisciplinary consensus (histological results for N2/3 LNs in 96%).

**Results:**

N2/3 disease was present in 190 patients (39%; training + test, 37%; validation, 46%; *p* = 0.09). A gradient boosting classifier (GBM) with 10 features was selected as the final model based on test AUC of 0.91 (95% confidence interval, 0.87–0.94). Validation AUC was 0.94 (0.89–0.98). At a target sensitivity of approx. 90%, test/validation accuracy of the GBM was 0.78/0.87. This was significantly higher than the accuracy based on “mediastinal LN uptake > mediastinum” (0.7/0.75; each *p* < 0.05) or combined PET/CT criteria (PET positive and/or LN short axis diameter > 10 mm; 0.68/0.75; each *p* < 0.001). Harmonization of PET images between the two scanners affected SUVmax and visual assessment of the LNs but did not diminish the AUC of the GBM.

**Conclusions:**

A machine learning model based on routinely available variables from [^18^F]FDG-PET/CT improved accuracy in mediastinal LN staging compared to established visual assessment criteria. A web application implementing this model was made available.

**Supplementary Information:**

The online version contains supplementary material available at 10.1007/s00259-023-06145-z.

## Introduction

In patients with non-small cell lung cancer (NSCLC), accurate pretherapeutic imaging-based assessment of thoracic lymph node (LN) metastases is essential for treatment planning. Most importantly, identification of metastatic spread to LNs in the N2 or N3 region is critical because patients with N0/1 disease are usually referred to surgery while those with N2/3 disease require multimodal treatment [1]. Positron emission tomography/computed tomography (PET/CT) with [^18^F]fluorodeoxyglucose (FDG) is the most accurate imaging modality for LN assessment in NSCLC. LNs are most commonly regarded as positive in [^18^F]FDG-PET/CT if their uptake is higher than the mediastinal background activity [2] or if their short axis diameter is > 10 mm [3–6]. However, when using these criteria, the desired high sensitivity comes with unacceptably low specificity. Gunluoglu et al. reported that > 90% of patients who were candidates for surgery required invasive confirmation of suspicious mediastinal LNs in [^18^F]FDG-PET/CT while only 26% of those cases were confirmed to be pN2/3 [7].

Several authors have employed machine learning methods to improve diagnostic accuracy. These have been based either on manually defined features such as LN size or on standardized uptake value (SUV) [8, 9], radiomic features [10, 11], or deep learning from segmented images [12]. In these reports, machine learning methods achieved an accuracy similar or superior to experienced physicians in determining LN status at the patient level [8, 12] or LN level [10, 11]. However, none of the models has been validated with a separate PET/CT scanner, none of these studies has compared accuracy with previously published models from other groups, and none of the models developed has been distributed as a ready-to-use diagnostic tool. Notably, if textural features such as radiomics or deep learning-derived features are employed, it is crucial to demonstrate the transferability of the model to other scanners. Furthermore, feature extraction or image segmentation may not be practicable in routine clinical care.

The aim of the current analysis was therefore to develop a diagnostic machine learning model to differentiate the clinically relevant categories N0/1 and N2/3 using variables from [^18^F]FDG-PET/CT images and clinical/pathological data that can be obtained in any patient with routinely available tools. Furthermore, the performance of our model was compared to that of one developed by Toney et al. [8] which achieved exceptionably high accuracy of 99.2% in differentiating NSCLC patients with N0/1 from N2/3 using simple primary tumor and LN metrics but which has not been validated externally. To facilitate further validation of our machine learning model, all variables and the machine learning code have been published as Open Data, and a user-friendly web application for use of our model is available online.

## Methods

### Patients

A flow diagram of patients is provided in Supplementary material #1 together with the Standards for the Reporting of Diagnostic Accuracy Studies (STARD) 2015 checklist.

One thousand and twenty-five consecutive patients with newly diagnosed NSCLC or with suspected lung cancer but unsuccessful biopsy attempts underwent [^18^F]FDG-PET/CT as part of routine clinical care at the same tertiary hospital between February 2013 and September 2020. Those fulfilling the following inclusion criteria were retrospectively identified for analysis: (i) histologically proven NSCLC, (ii) [^18^F]FDG-PET/CT performed before initiation of any treatment, (iii) blood glucose level ≤ 190 mg/dl, and (iv) histology of thoracic lymph node stations available between 6 weeks before to 8 weeks after [^18^F]FDG-PET/CT (but not necessarily including histology of N2/3 lymph nodes).

The 491 patients included in the analysis were divided into two separate cohorts based on two different PET/CT scanners which had been used to examine the patients. The training + test cohort, comprising 385 patients, was examined with a Philips Gemini TF scanner equipped with conventional photomultiplier tubes and time of flight (TOF) capability [13] between February 2013 and August 2020. The validation cohort, consisting of 106 patients, was scanned between February 2018 and September 2020 with a GE Discovery MI PET/CT with silicon photomultipliers (SiPM) and TOF capability [14].

All procedures were carried out in accordance with the local ethics commission (protocol #EA2/100/21).

### [^18^F]FDG-PET/CT protocol

Supplementary material #2 provides details on the [^18^F]FDG-PET/CT imaging and image reconstruction protocol. Briefly, PET/CT imaging in the training + test cohort was performed with a median injected [^18^F]FDG activity of 267 MBq and a median uptake time of 70 min. PET images were reconstructed iteratively with ordered subset expectation maximization and TOF. In the validation cohort, a median activity of 261 MBq [^18^F]FDG was administered, and the median uptake time was 65 min. PET raw data were reconstructed with a Bayesian penalized likelihood reconstruction algorithm (GE “Q.Clear,” beta = 450) [15].

### PET: visual assessment

Intensity of the lymph node [^18^F]FDG uptake was rated with a 4-step score as previously described [16]. The score was defined as follows: (1) LN uptake appears ≤ mediastinal background, (2) LN uptake > mediastinal background but < liver, (3) LN uptake ≥ liver but not “black,” and (4) LN appears “black.” PET window was usually set to a mean of 2.5 and a width of 5.0 (SUV). Visual assessment was conducted by one experienced nuclear medicine physician (JMMR, approx. 12 years of experience in [^18^F]FDG-PET/CT in lung cancer) using the Visage 7.1 viewer (version 7.1.17, Visage Imaging, Inc., San Diego, CA, USA). The reader was fully blinded to the results of the reference standard.

### PET: SUV measurements

The highest SUVmax (corrected for total body mass) in each LN region (N1, N2, N3) was measured using a 3D volume of interest (VOI) using the Visage 7.1 viewer. In addition, based on the methodology of Toney et al. [8], lung background SUVmean was calculated as the average SUVmean of circular 2D regions of interest (ROIs) placed in three subsequent images slices in the normal lung parenchyma contralateral to the primary tumor (Supplementary material #2). Similarly, the mediastinal background SUVmean was determined using 2D ROIs in three subsequent image slices in the right pulmonary artery (below the carina). As described by Toney et al. [8], SUV ratios were derived for each LN region. LN SUVmax was either divided by the lung background SUVmean (N1) or mediastinal background SUVmean (N2, N3). If a LN region was considered PET negative (uptake ≤ mediastinal background; i.e., visual score = 1), the background SUVmean was used instead of the aforementioned SUV ratio. Again, lung background SUVmean was used for the N1 region and the mediastinum background SUVmean for N2 and N3. In line with [8], LN SUVs were not corrected for partial volume effect.

Primary tumor SUVmax was measured with a 3D VOI using the Visage 7 software. In line with Toney et al. [8] and as previously reported by those authors [17], partial volume correction was performed here (Supplementary material #2).

### CT assessment

LN short axis diameters and primary tumor diameter (largest tumor diameter in transaxial plane in the lung window) were measured using contrast-enhanced CT data if available (obtained as part of the [^18^F]FDG-PET/CT or within 6 weeks of [^18^F]FDG-PET/CT). In 66 patients (13%), only non-enhanced CT data were available. One LN short axis diameter was measured for the N1, N2, and N3 regions, respectively. In each region, the LN with the highest short axis diameter was taken as decisive, irrespective of its [^18^F]FDG accumulation.

### Clinical and pathological data

Clinical and pathological data were collected retrospectively from the hospital information system and tumor documentation system. This included demographic data (age, sex), tumor characteristics (affected side and lobe, central vs. peripheral growth, c/pT stage), histological data (NSCLC subtype, grade of differentiation), smoking behavior (never vs. former vs. current, pack years), work-related exposure to inhalable toxins, acute inflammatory pulmonary disease (e.g., pneumonia), structural pulmonary disease (e.g., emphysema or fibrosis), and use of immunosuppressive medication or presence of immunosuppressive disease.

A full list of features from [^18^F]FDG-PET/CT and clinical/pathological data can be found in Supplementary material #3. More detailed descriptions of each feature and its categories are provided in the Open Data repository zenodo (10.5281/zenodo.7094287).

### Reference standard

The reference standard used to evaluate model performance was the final N stage determined by interdisciplinary consensus as part of the tumor board decision. In 351 of the 491 patients analyzed (71%), this was based on surgery with systematic hilar and mediastinal lymph node resection. The remaining 140 patients (29%) did not undergo surgery of the primary tumor. In these patients, N stage was either determined through LN biopsy (usually EBUS-guided transbronchial needle biopsy with punch cylinders that enabled histological examination) in 122 patients (25%) or based on EBUS-TBNA of the N1 station (in 18 patients (4%) with extensive locoregional or metastatic disease where biopsy confirmation of unequivocal imaging results regarding N2/3 LNs was deemed unnecessary). Patients without surgery were not excluded because the ideal machine learning model should be applicable to all patients with NSCLC who undergo [^18^F]FDG-PET/CT prior to potentially curative treatment.

### Machine learning

All scripts that were used for the following steps of feature preprocessing and machine learning are provided in the Open Data repository zenodo (10.5281/zenodo.7094287).

#### Feature preprocessing

Missing values were present in 6 of the 40 variables with a percentage of missing values between 0.4% (T stage) and 21% (histological grade of differentiation). These missing values were imputed (details in Supplementary material #3). Categorical variables were subsequently one-hot encoded, resulting in a final list of 80 (dummy) variables (full list in Supplementary material #3).

#### Model training, testing, and validation

Five different machine learning methods were used for training with tenfold nested cross-validation: a random forest classifier (RF), support vector classifier (SVC), gradient boosting classifier (GBM), XGBoost classifier (XGB), and a multi-layer perceptron classifier (MLP). The training + test cohort was split into training and test sets using 10 folds (“outer loop”). Within each training set (“inner loop”), an *sklearn pipeline* was created to integrate robust scaling, feature selection, and hyperparameter tuning (grid search) in 10 folds.

In this *pipeline*, feature selection used one of the following five methods (mutual information classifier, RF, linear SVC, GBM, and an AdaBoost classifier). The maximum number of variables to remain after feature selection was set at *n* = 30. To evaluate whether a lower number of features would yield similar model performance in the test folds, *n* = 25, *n* = 20, *n* = 15, *n* = 10, *n* = 5, and *n* = 1 features were also investigated. In total, this resulted in 5 * 7 = 35 different sets of selected features.

Details of the grid of hyperparameters searched for each model can be found in Supplementary material #3.

The aim of the training was maximizing the AUC in receiver operating characteristics (ROC) analysis for the binary classification into N0/1 vs. N2/3. The highest mean AUC of all 10 test folds was used to identify the best model. In addition, all trained models were fitted to the whole training + test dataset and validated using the validation cohort, which was set aside as a hold-out dataset.

Having identified the best combination of feature selection (GBM, *n* = 10 features) and estimator/classifier (GBM), it was investigated if random oversampling of the minority class (N2/3) would further improve the AUC by adjusting the imbalance in the dataset. The RandomOverSampler from the *Imbalanced-Learn* package for *python* was used for this task. The full hyperparameter space for the GBM was searched.

#### Alternative model design (Toney et al.)

Based on the publication of Toney et al. [8], a feed-forward multilayer ANN with three layers (input, hidden layer, output) was constructed with the *nnet* package in R × 64 4.2.1 (The R Project for Statistical Computing) and the following parameters: maxit = 1000; size = 8; skip = True; softmax = False; decay = 0; rang = 0. Like the aforementioned models, the ANN was used for binary classification into N0/1 vs. N2/3. This was done to ensure that the model performances in the current analysis could be compared (Toney et al. also reported results for N0 vs. N1 vs. N2 vs. N3).

Model training used the training + test cohort, which was randomly split into two equally sized sets, as described by Toney et al. [8]. One set was used for model training, the second for testing (AUC). This procedure of data splitting and model training was repeated 100 times to obtain a mean test AUC. For each of the 100 training sets, the model weights were also used to calculate a mean AUC for the hold-out validation set.

### Validation cohort: retrospective smoothing of PET data

Both visual assessment and SUV measurements from PET images are potentially affected by image reconstruction. Robustness of the results from the visual assessment and the machine learning model regarding different image characteristics was therefore investigated in the validation cohort. For this purpose, the originally reconstructed PET data were smoothed with an additional Gaussian filter [18], which resulted in a reconstructed spatial resolution similar to that of the training + test cohort scanner (details in Supplementary material #2). SUVmax of the primary tumor and the N1, N2, and N3 LN region were measured again—blinded to the results of the original measurements and to the reference standard. Furthermore, the visual PET score of the mediastinal LNs was obtained again. Background SUVmean were assumed to be equal to the original PET data [19] and therefore not re-analyzed. Using these values, validation of the final machine learning model was performed again to assess the impact of smoothing the PET data on model performance in the validation cohort (model training and testing were not affected).

### Statistical analysis

Data collection was performed with SPSS 28 (IBM, Armonk, NY, USA). Non-parametric data distribution was assumed, based on the Shapiro–Wilk test, and median values with interquartile range (IQR) and range were used for descriptive analysis. Patient characteristics were compared between the two cohorts with the two-sided Fisher’s exact test or Wilcoxon rank-sum test. Model performance was evaluated with AUCs and corresponding 95% confidence intervals (95% CI) from ROC analysis in SPSS.

To calculate the AUC ± SD of the final model, the predicted probabilities for all patients in the training + test cohort were calculated from the 10 test folds during cross-validation (*cross_val_predict* function from *sklearn*). Predicted probabilities for the validation cohort were calculated after refitting the final model to the entire training + test cohort.

Likewise, the AUC ± SD of the ANN by Toney et al. was derived from the predicted probabilities. However, in line with Toney et al. who originally proposed a 1:1 split of training and test data with 100 repeats, predicted probabilities for the training + test cohort were the mean probabilities of these 100 repeats. Validation AUC ± SD was again obtained from the refitted model.

The method by DeLong et al. [20] was used for the pairwise comparison of these model AUCs and the visual PET score (MedCalc version 15.8, MedCalc Software Ltd, Ostend, Belgium). AUC was chosen as the primary parameter to evaluate model performance to avoid bias from imbalanced data arising from the fact that cases with N2/3 were less frequent than N0/1 cases. Diagnostic accuracy was compared using McNemar’s test in SPSS.

In the validation cohort, SUVmax of original and smoothed PET data were compared with the Wilcoxon signed-rank test. Visual PET scores were compared with the McNemar-Bowker test. AUCs were compared using the method by DeLong et al. Statistical significance was assumed at *α* = 0.05.

## Results

### Reference standard

In the training + test cohort, 141 of 385 patients (37%) were N2/3. This was slightly lower than the percentage of patients with N2/3 (46%) in the validation cohort (Fisher’s exact test, *p* = 0.09). Other patient characteristics are shown in Table 1.

**Table 1:** Patient characteristics

Parameter	Total	Training + test	Validation	*p*-value
Patient count	491	385	106	–-
Age (years)	68 (60–75)	68 (60–75)	68 (60–75)	0.85
Sex: male	300 (61)	247 (64)	53 (50)	**0.01**
T stage				0.55
T1	165 (34)	133 (35)	32 (30)	
T2	166 (34)	125 (32)	41 (39)	
T3	97 (20)	79 (21)	18 (17)	
T4	63 (13)	48 (10)	15 (14)	
N stage				0.19
N0	248 (51)	200 (52)	48 (45)	
N1	53 (11)	44 (11)	9 (8)	
N2	129 (26)	99 (26)	30 (28)	
N3	61 (12)	42 (9)	19 (18)	
N2/3	190 (39)	141 (37)	49 (46)	0.09
Type of reference standard				0.11
Surgery	351 (71)	283 (74)	68 (64)	
EBUS-TBNA including N2/3 LNs	122 (25)	90 (23)	32 (30)	
EBUS-TBNA of N1 LNs + unequivocal imaging	18 (4)	12 (3)	6 (6)	
Primary tumor size (mm)	32 (21–51)	34 (22–52)	30 (20–46)	0.088
Primary tumor SUVmax	12.2 (7.7–17.1)	12.1 (7.5–17.3)	12.8 (8.3–16.6)	0.42
NSCLC subtype				0.19
ADC	281 (57)	217 (56)	64 (60)	
SCC	165 (34)	136 (35)	29 (27)	
Others/NOS	45 (9)	32 (8)	13 (12)	
Smoking behavior				0.88
Never	43 (9)	35 (9)	8 (8)	
Former	223 (45)	173 (44)	50 (47)	
Current	225 (46)	177 (46)	48 (45)	
Acute inflammatory lung disease				1.0
Absent	467 (95)	366 (95)	101 (95)	
Present	24 (5)	19 (5)	5 (5)	

Results are given as count (%) or median (IQR), respectively. *P*-values are either from two-sided Fisher’s exact test or Wilcoxon rank-sum test. Significant results (i.e., *p* < 0.05) are highlighted in bold

*ADC* adenocarcinoma, *SCC* squamous cell carcinoma, *NOS* not otherwise specified, *LNs*, lymph nodes

### Diagnostic performance: machine learning models

**Fig. 1:**
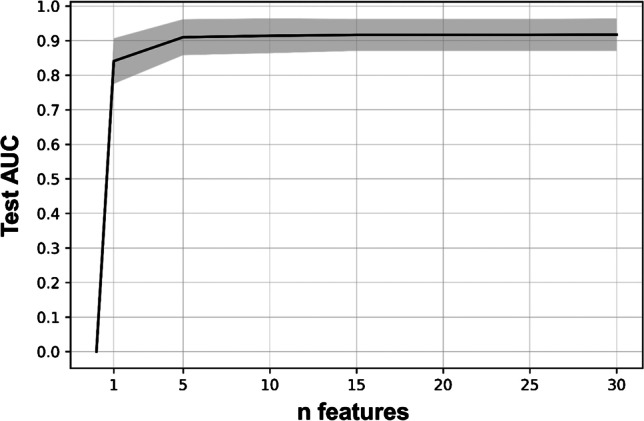
Relationship between feature count and test AUC. Mean test AUC of the GBM among all the 10 test folds is displayed with its standard deviation highlighted in gray

A GBM showed the highest mean test AUC at 0.91 (95% CI, 0.87 to 0.94; Table 2) and the highest validation AUC at 0.94 (0.89 to 0.98; Table 3). It contained *n* = 10 features (Table 4), which were identified during feature selection with a preceding GBM. Increasing the number of features did not further improve the AUC (Fig. 1). Other models achieved a similar test AUC but required a higher feature count and were therefore rejected (Supplementary material #4).

**Table 2:** Diagnostic performance of different models in the training + test cohort

Model	Test AUC	Sensitivity	Specificity	Accuracy
*Machine learning*				
Final model: GBM	0.91 (0.87–0.94)	–-	–-	–-
Probability > 0.5	–-	0.73 (0.65–0.8)	0.95 (0.91–0.97)	0.87 (0.83–0.9)
Probability > 0.19	–-	0.9 (0.84–0.94)	0.7 (0.64–0.76)	0.78 (0.73–0.82)
ANN (Toney et al.)	0.9 (0.86–0.93)	–-	–-	–-
Probability > 0.5	–-	0.73 (0.65–0.8)	0.94 (0.91–0.97)	0.86 (0.83–0.9)
Probability > 0.12	–-	0.9 (0.84–0.94)	0.7 (0.63–0.75)	0.77 (0.73–0.81)
*Visual assessment (only mediastinal LNs decisive)*				
Visual PET score	0.87 (0.83–0.91)	–-	–-	–-
≥ 2	–-	0.89 (0.82–0.93)	0.59 (0.53–0.65)	0.7 (0.65–0.74)
PET score ≥ 2 and/or LN size > 10 mm	–-	0.92 (0.86–0.96)	0.53 (0.47–0.6)	0.68 (0.63–0.72)

Results in parentheses are 95% confidence intervals. PET score ≥ 2 indicates that uptake by the LN is higher than the mediastinal background. LN size was the short axis diameter

**Table 3:** Diagnostic performance of different models in the validation cohort

Model	Validation AUC	Sensitivity	Specificity	Accuracy
*Machine learning*				
Final model: GBM	0.94 (0.89-0.98)	---	---	---
probability >0.5	---	0.88 (0.75-0.95)	0.91 (0.81-0.97)	0.9 (0.82-0.95)
probability >0.19	---	0.92 (0.8-0.98)	0.82 (0.7-0.91)	0.87 (0.79-0.93)
ANN (Toney *et al.*)	0.78 (0.68-0.87)	---	---	---
probability >0.5	---	0.67 (0.52-0.8)	0.88 (0.76-0.95)	0.78 (0.69-0.86)
probability >0.12	---	0.67 (0.52-0.8)	0.88 (0.76-0.95)	0.78 (0.69-0.86)
*Visual assessment (only mediastinal LNs decisive)*				
Visual PET score	0.91 (0.85-0.97)	---	---	---
≥2	---	0.96 (0.86-1.0)	0.58 (0.44-0.71)	0.75 (0.66-0.83)
PET score ≥2 and/ or LN size >10 mm	---	0.96 (0.86-1.0)	0.58 (0.44-0.71)	0.75 (0.66-0.83)
*Smoothed PET data*				
Final model: GBM	0.94 (0.89-0.99)	---	---	---
probability >0.19	---	0.9 (0.78-0.97)	0.84 (0.72-0.93)	0.87 (0.79-0.93)
PET score ≥2 and/ or LN size >10 mm	---	0.96 (0.86-1.0)	0.61 (0.48-0.74)	0.77 (0.68-0.85)

Results in parentheses are 95% confidence intervals. PET score ≥2 means that uptake by the LN is higher than the mediastinal background. LN size was the short axis diameter. Results from the retrospectively smoothed PET data (lower segment of the table) were similar to those obtained from the original PET data.

**Table 4:** List of the 10 features in the final GBM model

Feature (or dummy variable)	Frequency (%)
N1 LN SUVmax (non-corrected)	100
N2 LN SUVmax (non-corrected)	100
Toney et al.: N1 LN short axis diameter (mm)	100
Toney et al.: N2 LN short axis diameter (mm)	98
Visual PET score (mediastinal LNs) = 4: *yes* vs. *no*	95
Age (years)	90
Toney et al.: Primary tumor diameter (mm)	88
Toney et al.: N1 LN SUVmax (background-corrected)	71
Toney et al.: N2 LN SUVmax (background-corrected)	68
Toney et al.: N3 LN SUVmax (background-corrected)	66

These variables were selected through tenfold nested cross-validation (= 100 folds in total) using a separate GBM for the feature selection step. Frequency was calculated as the sum of all folds in which the variable ranked among the top 10 most important features. Hence, this frequency reflects relative feature importance (optimum: 100)

The mean test AUC of 0.91 for the GBM was comparable to that of the ANN by Toney et al. which achieved a test AUC of 0.9 (DeLong test, *p* = 0.45). In the validation cohort, the AUC of the GBM was significantly higher than that of the ANN (0.94 vs. 0.78; *p* < 0.0001).

Random oversampling of the N2/3 class did not improve the model performance of the GBM compared to the original dataset (test AUC, 0.9 (0.87–0.94); validation AUC, 0.91 (0.85–0.98)).

### Diagnostic performance: standardized visual assessment

To assess the added value of machine learning, a comparison was made with standardized visual assessment using established diagnostic criteria (Tables 2 and 3). The most common criterion for PET-positive LNs is uptake higher than the mediastinal background (i.e., a visual score ≥ 2 in the current analysis). A reader could rely on the mediastinal LNs alone (given that where only contralateral hilar LNs are suspicious, this is most likely to be a false positive) or contralateral hilar LNs could be taken as decisive as well. The latter approach would have been less specific (Supplementary material #4).

The GBM model showed significantly higher AUC than the visual PET score in the training + test cohort (0.91 vs. 0.87; *p* = 0.003) and slightly higher AUC in the validation cohort (0.94 vs. 0.91; *p* = 0.23).

**Fig. 2:**
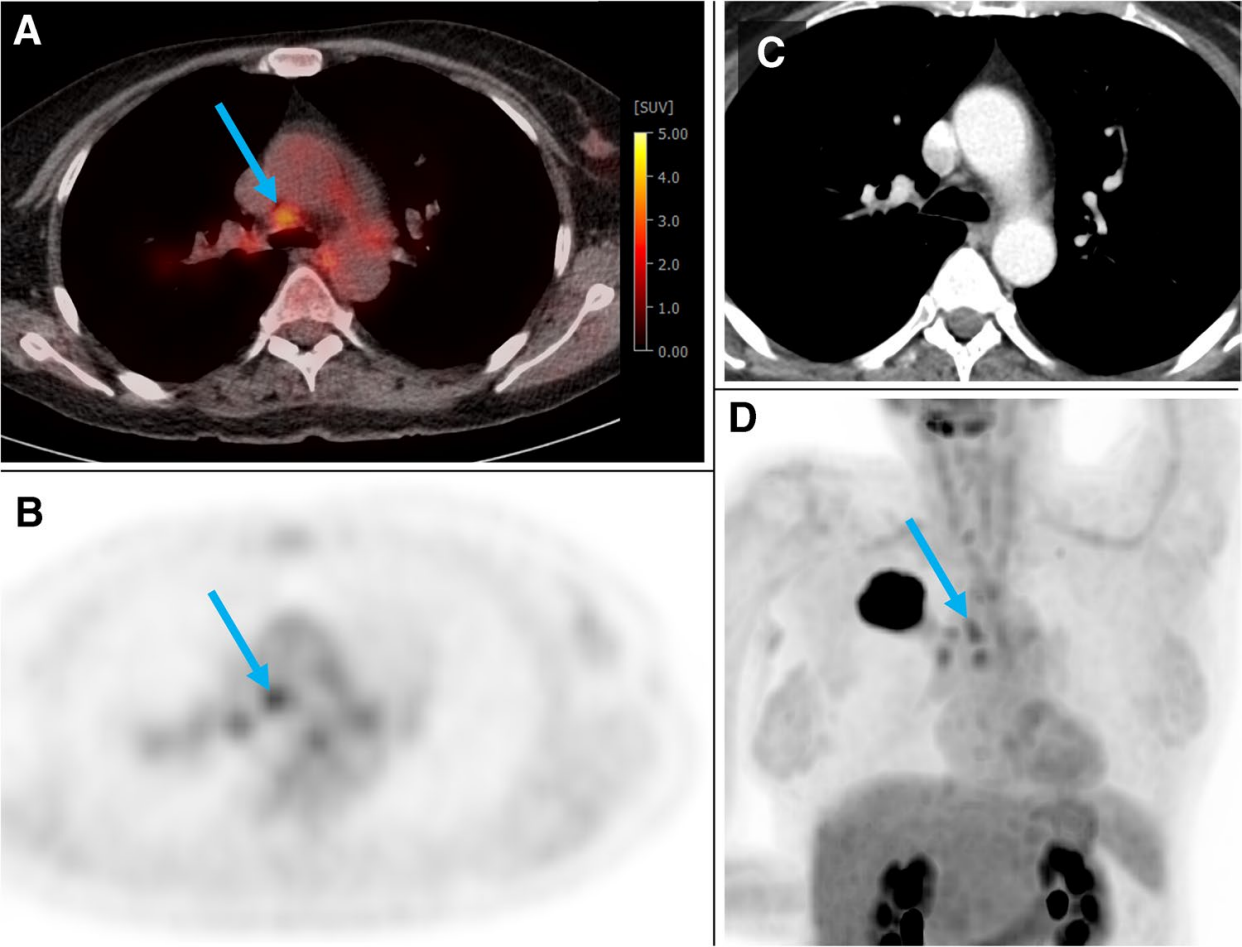
Case example #1**.** Fifty-four-year-old woman from the training + test cohort with a G2 adenocarcinoma of the right upper lobe (44 mm). Several hilar and mediastinal lymph nodes (LN) showed higher [^18^F]FDG uptake than the normal liver (= PET score of 3).The blue arrow depicts an N2 paratracheal LN with 7 mm short axis and SUVmax of 3.5. Based on visual assessment, N2 disease would be suspected in this case. According to the GBM model, probability of N2/3 is only 0.17 (= negative). The patient was confirmed to be N0 by ultrasound-guided transbronchial needle biopsy of the ipsilateral hilar, paratracheal and subcarinal LNs

At a clinically useful sensitivity of approx. 90%, the accuracy of the GBM was significantly higher than that of a visual PET score ≥ 2 or a combined PET/CT approach (PET score ≥ 2 and/or LN size > 10 mm) in the training + test cohort and the validation cohort (McNemar’s test, each *p* < 0.05). Figure 2 shows a case example, and Supplementary material #4 contains three more cases with discordant results of visual assessment vs. the GBM.

### Diagnostic performance: harmonized (smoothed) PET data

In the validation cohort, LN SUVmax (without background correction) in the smoothed PET data were significantly lower than with the original PET data by a median of approx. 23% (N1: median, − 22% (IQR, − 29 to − 16%); N2: 23% (− 30 to − 18%); N3: − 23% (− 28 to − 18%); Wilcoxon signed-rank test, each *p* < 0.001). Scatterplots are shown in Supplementary material #4, Figure S4.8. Visual PET scores of the mediastinal LNs were lower in 12 of 106 patients (11%; Supplementary material #4, Table S4.9) and were unchanged in the remaining 94 patients (McNemar-Bowker test, *p* = 0.022).

Using these results, predicted probabilities of the GBM in the validation cohort were significantly lower with a small median absolute difference of − 0.0015 (IQR, − 0.03 to 0.0; Wilcoxon signed-rank test, *p* < 0.001; Supplementary material #4, Figure S4.10). However, the AUC of the GBM in the validation cohort (AUC, 0.94; 95% confidence interval, 0.89–0.99) remained unchanged compared to the AUC from the original PET data (0.94 (0.89–0.98); DeLong test, *p* = 0.96; Table 3). Compared to the original data, two of 106 patients (2%) were classified discordantly by the GBM when applying the threshold > 0.19 (McNemar’s test, *p* = 0.5). Of these two patients, one patient with pN0 was classified correctly based on the smoothed PET data (true negative), while one patient with pN2 was classified incorrectly (false negative).

Illustrative case examples of the original vs. smoothed PET data are shown in the case examples in the Supplementary material #4.

### Web application

An openly accessible web application was programmed and published at GitHub (https://baumgagl.github.io/PET_LN_calculator/). It takes user input of 10 features to calculate the predicted probability of N2/3 disease based on the final GBM model.

## Discussion

This analysis shows that machine learning can improve diagnostic accuracy in pretherapeutic LN staging of patients with NSCLC. Our GBM model was superior to LN uptake above the mediastinum (i.e., a visual PET score ≥ 2), which is the most common and validated diagnostic threshold in visual [^18^F]FDG-PET/CT assessment [2]. An important clinical goal is to achieve a sensitivity of 0.9. In its 2014 guidelines for preoperative LN staging in NSCLC, the European Society of Thoracic Surgeons (ESTS) states that—while aiming for optimal accuracy—a 10% rate of unforeseen pN2 could be considered acceptable, given that preoperatively undetected pN2 is usually single-level and resectable. [21]. At this sensitivity level, the GBM was more accurate than visual PET or PET/CT assessment based on the mediastinum threshold.

An expert might have achieved higher specificity than such rigid criteria by recognizing conditions or patterns of false positive imaging findings (e.g., multiple LNs with symmetrically increased FDG uptake). However, this formalized approach was chosen to ensure transparency and reproducibility of the diagnostic criteria and threshold. This visual PET score has proven to be of high diagnostic accuracy on the level of individual LN stations [16, 22, 23]. Moreover, in a previous study, inter-reader agreement in assigning the standardized visual PET score was high with kappa > 0.9 between unexperienced and experienced readers [16]. Application of the full machine learning model might also be possible with the sufficient inter-reader agreement—although this requires confirmation.

It may be noted that the visual PET score is subject to inter-scanner differences. PET scanners or image reconstruction algorithms with better reconstructed spatial resolution will tend to show more intense focal uptake of LNs and therefore a potentially higher visual PET score. Likewise, and probably to a larger extent, SUVmax are higher if the reconstructed spatial resolution is better [24, 25]. In the current analysis, we retrospectively smoothed the PET data from the validation cohort scanner to simulate how SUVmax and visual PET scores would have been if the reconstructed spatial resolution in the validation cohort had been similar to that of the training + test cohort scanner. It has been shown before that this approach effectively harmonizes SUVmax between scanners [25] and facilitates the application of prognostic SUVmax thresholds in multicentre settings [26]. In the present analysis, smoothing the PET data significantly decreased LN SUVmax and visual PET scores. However, although this decreased predicted probabilities of the GBM on average, this did not affect the performance of the model at the threshold of > 0.19. This suggests that its performance is relatively robust against different PET image characteristics. This observation may be explained by the variety of features that are part of the model, four of which are not affected by visual or SUV analysis of the PET images.

Among the ten features in the final GBM (Table 4), LN SUVmax and short axis diameters as well as the visual PET score are best supported by previous studies [2, 16, 22]. Background-corrected LN SUVmax could provide added value because they may decrease inter-patient SUV variability and accentuate SUV differences between visually unremarkable LNs and those with increased [^18^F]FDG uptake. The uptake (SUVmax) of N1 LNs seems to be important to increase diagnostic sensitivity for N2/3 disease because all true positive cases with the GBM that were false negative by visual assessment showed high N1 LN uptake (visual PET score = 4). The association between primary tumor size and the risk of undetected N2/3 disease has been demonstrated before [27]. Our observation that the probability of N2/3 decreases with higher age is in line with an analysis of the Surveillance, Epidemiology, and End Results (SEER) database [28].

The model developed here is intended for use in routine clinical care to estimate the probability of N2/3 disease based on [^18^F]FDG-PET/CT findings and to form a basis for interdisciplinary decisions on the need for further invasive diagnostic procedures. If a target sensitivity of 90% is deemed adequate, patients with a model-based predicted probability of ≤ 0.19 would not require pretherapeutic invasive staging. To facilitate the usage of the model, a user-friendly online tool can be accessed (https://baumgagl.github.io/PET_LN_calculator/).

Performance levels did not greatly differ between multiple feature selection methods and different models, and only a limited number of features *n* < 10 considerably affected model AUC (Supplementary material #4, Table S4.3). This is due to the exceptionally high feature importance of the top 10 variables (Table 4). Other parameters that were included with the aim of increasing specificity, such as smoking behavior, structural pulmonary disease, or acute inflammatory lung disease, did not contribute to model performance.

Performance of the GBM and the visual PET criteria were slightly better in the validation cohort than in the training + test cohort. Diagnostic sensitivity is potentially higher with the newer PET/CT scanner and image reconstruction algorithm used in the validation cohort, especially in smaller lesions [29].

Among previous reports on the use of machine learning models for LN staging in NSCLC, only the methodology set out by Toney et al. [8] can be reproduced in a different cohort with relative ease, as all the variables are routinely obtainable. Minor deviations in the current methodology compared to the authors’ original publication should be noted. Toney et al. determined the highest short axis LN diameter for each region from the “hottest” LN in PET whereas, in the current analysis, the highest short axis LN diameter was determined independently from the [^18^F]FDG uptake. To accelerate measurements, we measured only one transaxial primary tumor diameter instead of taking the average of all three lesion diameters. Furthermore, Toney et al. measured the mediastinum SUVmean “a few mm below the carina.” As this localization is prone to high interindividual differences in tissue composition (blood pool vs. pericardial fat tissue vs. subcarinal LN tissue), we decided to determine this SUVmean uniformly from the blood pool in the right pulmonary artery.

In both cohorts studied here, the ANN by Toney et al. was inferior to the GBM and to the results in their original publication [8]. Notably, the originally reported accuracy of 99.2% in 133 patients was exceptionally high considering that in sufficiently large cohorts, accuracy of [^18^F]FDG-PET/CT for LN staging in NSCLC rarely exceeds 90% [2]. Possible explanations for these deviating results are the lack of an external/independent validation cohort in the original publication and the fact that the model was first developed based on stand-alone [^18^F]FDG-PET images with image reconstruction using transmission scan-based attenuation correction and filtered back projection. SUV from such image data are not comparable with current scanners.

Although the results analyzed here came from two very different PET/CT scanners, all patients were examined and treated at the same hospital. The use of two types of scanner will have served to increase the generalizability of the image features that were part of the final model. This is underlined by the observation that the performance of the GBM in the validation cohort was unaffected by smoothing that was retrospectively applied to the PET data. The fact that histological proof of the N2/3 status was not available in all patients should have prevented selection bias from thr exclusion of patients with advanced disease. External validation of our findings is pending. It could also be worth investigating the SUVpeak as an alternative to the SUVmax, given that the SUVpeak is more comparable between different reconstruction algorithms [30], and its test–retest repeatability is better [31].

## Conclusion

The machine learning model that was developed in this work improved accuracy in pretherapeutic mediastinal LN staging for NSCLC compared to established visual [^18^F]FDG-PET/CT assessment criteria. It is based on routinely available variables, the majority of which are already part of [^18^F]FDG-PET/CT reporting in routine clinical care. To facilitate its use, a web application implementing this model was made available. The observation of a high AUC in the validation cohort and that smoothing of PET images in the validation cohort did not diminish the performance of the model suggests that it could provide good generalizability to other PET scanners. However, external validation and proof of its validity in an interventional trial are pending.

## Supplementary Information

Below is the link to the electronic supplementary material.

Supplementary file1 (PDF 884 KB)

Supplementary file2 (PDF 578 KB)

Supplementary file3 (PDF 338 KB)

Supplementary file4 (PDF 990 KB)

## Data Availability

The datasets and scripts generated during and/or analysed during the current study are available in the zenodo repository, 10.5281/zenodo.7094287. However, it only contains a limited dataset with the 10 features that are required to build the final GBM model. The original full dataset with all 40 variables was excluded from the Open Data upload because it may not fully comply with strict requirements for anonymized data. This full dataset, which allows to reproduce all results, can be obtained from the corresponding author upon reasonable request.
