# Supplementary material for: A machine learning tool to improve prediction of mediastinal lymph node metastases in non-small cell lung cancer using routinely obtainable [18F]FDG-PET/CT parameters
Source: Eur J Nucl Med Mol Imaging. 2023 Feb 23;50(7):2140–51. doi: 10.1007/s00259-023-06145-z (PMC10199849; doi:10.1007/s00259-023-06145-z)
Supplement: Supplementary file 1 — Supplementary file1 (PDF 884 KB) [file 259_2023_6145_MOESM1_ESM.pdf]

Supplementary material #1

Article title: A machine learning tool to improve prediction of mediastinal lymph node metastases in non-small cell lung cancer using routinely obtainable [^{18}F]FDG-PET/CT parameters

Journal name: European Journal of Nuclear Medicine and Molecular Imaging

Author names: Julian M.M. Rogasch, Liza Michaels, Georg L. Baumgärtner, Nikolaj Frost, Jens-Carsten Rückert, Jens Neudecker, Sebastian Ochsenreither, Manuela Gerhold, Bernd Schmidt, Paul Schneider, Holger Amthauer, Christian Furth, Tobias Penzkofer

Corresponding author:

Julian M.M. Rogasch

Department of Nuclear Medicine

Charité – Universitätsmedizin Berlin

Augustenburger Platz 1

D-13353 Berlin, Germany

Phone: +49 30 450 627106

Fax: +49 30 450 7557338

e-mail: julian.rogasch@charite.de

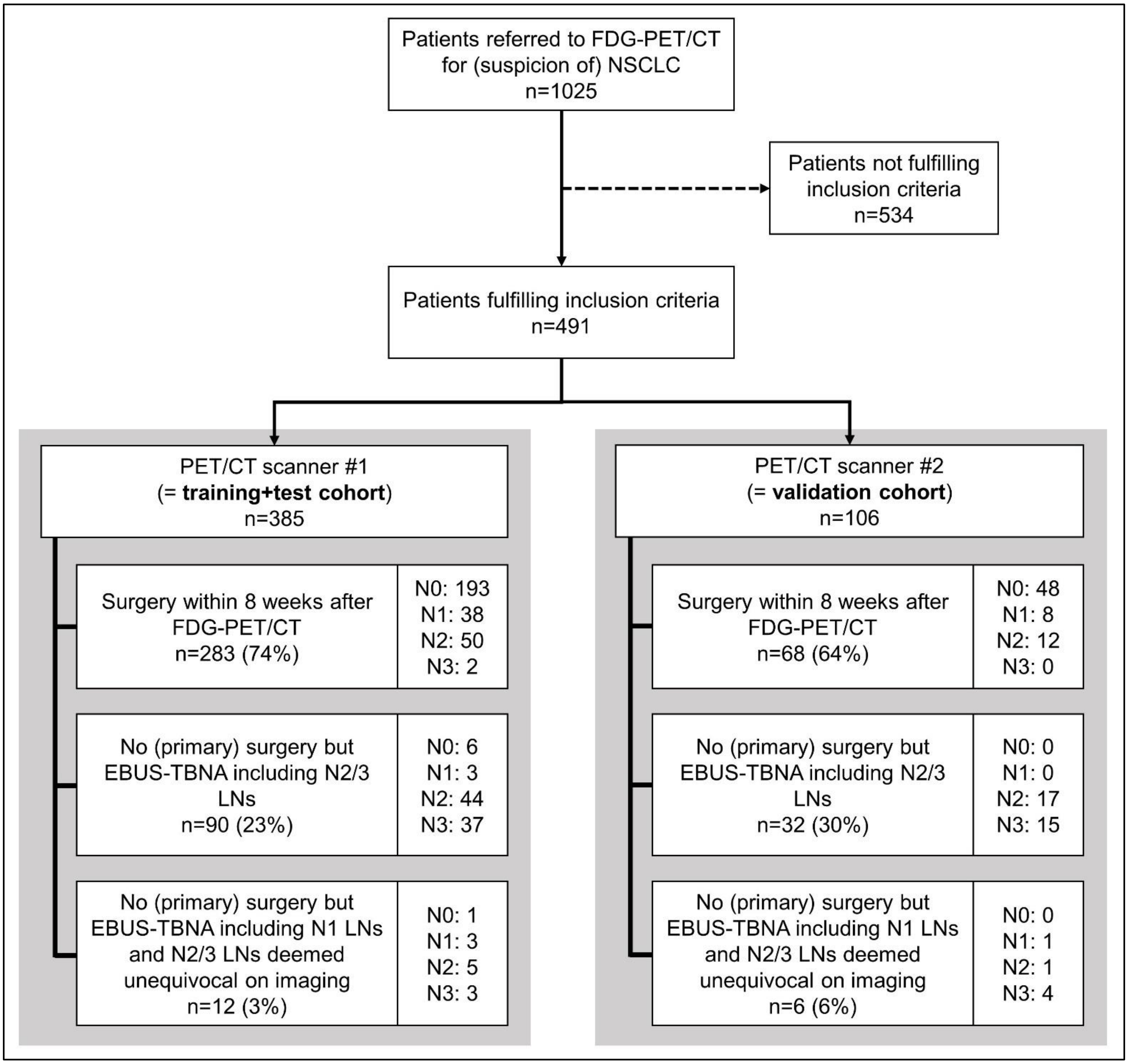

Figure S1.1. Flow diagram of patients in this analysis

LNs, lymph nodes.

STARD 2015 checklist

Section & Topic	No	Item	Reported on page #
**TITLE OR ABSTRACT**			
	**1**	Identification as a study of diagnostic accuracy using at least one measure of accuracy (such as sensitivity, specificity, predictive values, or AUC)	9/10
**ABSTRACT**			
	**2**	Structured summary of study design, methods, results, and conclusions (for specific guidance, see STARD for Abstracts)	2
**INTRODUCTION**			
	**3**	Scientific and clinical background, including the intended use and clinical role of the index test	3/4
	**4**	Study objectives and hypotheses	3/4
**METHODS**			
Study design	**5**	Whether data collection was planned before the index test and reference standard were performed (prospective study) or after (retrospective study)	5
Participants	**6**	Eligibility criteria	5
	**7**	On what basis potentially eligible participants were identified (such as symptoms, results from previous tests, inclusion in registry)	5
	**8**	Where and when potentially eligible participants were identified (setting, location and dates)	5
	**9**	Whether participants formed a consecutive, random or convenience series	5
Test methods	**10a**	Index test, in sufficient detail to allow replication	6 + multiple pages
	**10b**	Reference standard, in sufficient detail to allow replication	7
	**11**	Rationale for choosing the reference standard (if alternatives exist)	7/8
	**12a**	Definition of and rationale for test positivity cut-offs or result categories of the index test, distinguishing pre-specified from exploratory	Multiple pages
	**12b**	Definition of and rationale for test positivity cut-offs or result categories of the reference standard, distinguishing pre-specified from exploratory	7
	**13a**	Whether clinical information and reference standard results were available to the performers/readers of the index test	6
	**13b**	Whether clinical information and index test results were available to the assessors of the reference standard	They were not available
Analysis	**14**	Methods for estimating or comparing measures of diagnostic accuracy	9/10
	**15**	How indeterminate index test or reference standard results were handled	n/a
	**16**	How missing data on the index test and reference standard were handled	5 (eligibility criteria)
	**17**	Any analyses of variability in diagnostic accuracy, distinguishing pre-specified from exploratory	9/10 + tables
	**18**	Intended sample size and how it was determined	n/a
**RESULTS**			
Participants	**19**	Flow of participants, using a diagram	Above this table
	**20**	Baseline demographic and clinical characteristics of participants	Table 1
	**21a**	Distribution of severity of disease in those with the target condition	n/a
	**21b**	Distribution of alternative diagnoses in those without the target condition	n/a
	**22**	Time interval and any clinical interventions between index test and reference standard	n/a (retrospective)
Test results	**23**	Cross tabulation of the index test results (or their distribution) by the results of the reference standard	Table 2+3
	**24**	Estimates of diagnostic accuracy and their precision (such as 95% confidence intervals)	Results section
	**25**	Any adverse events from performing the index test or the reference standard	n/a
**DISCUSSION**			
	**26**	Study limitations, including sources of potential bias, statistical uncertainty, and generalisability	19
	**27**	Implications for practice, including the intended use and clinical role of the index test	17
**OTHER INFORMATION**			
	**28**	Registration number and name of registry	n/a
	**29**	Where the full study protocol can be accessed	n/a
	**30**	Sources of funding and other support; role of funders	none
