# Supplementary material for: A machine learning tool to improve prediction of mediastinal lymph node metastases in non-small cell lung cancer using routinely obtainable [18F]FDG-PET/CT parameters
Source: Eur J Nucl Med Mol Imaging. 2023 Feb 23;50(7):2140–51. doi: 10.1007/s00259-023-06145-z (PMC10199849; doi:10.1007/s00259-023-06145-z)
Supplement: Supplementary file 2 — Supplementary file2 (PDF 578 KB) [file 259_2023_6145_MOESM2_ESM.pdf]

# Supplementary material #2

**Article title:** A machine learning tool to improve prediction of mediastinal lymph node metastases in non-small cell lung cancer using routinely obtainable [$^{18}$F]FDG-PET/CT parameters

**Journal name:** European Journal of Nuclear Medicine and Molecular Imaging

**Author names:** Julian M.M. Rogasch, Liza Michaels, Georg L. Baumgärtner, Nikolaj Frost, Jens-Carsten Rückert, Jens Neudecker, Sebastian Ochsenreither, Manuela Gerhold, Bernd Schmidt, Paul Schneider, Holger Amthauer, Christian Furth, Tobias Penzkofer

Corresponding author:

Julian M.M. Rogasch
Department of Nuclear Medicine
Charité – Universitätsmedizin Berlin
Augustenburger Platz 1
D-13353 Berlin, Germany
Phone: +49 30 450 627106
Fax: +49 30 450 7557338
e-mail: julian.rogasch@charite.de

This Supplementary material #2 contains details on [$^{18}$F]FDG-PET/CT image acquisition and reconstruction as well as image assessment. The method for smoothing PET data from the validation cohort scanner is also described.

## [$^{18}$F]FDG-PET/CT imaging protocol

In the training+test cohort, [$^{18}$F]FDG-PET/CT imaging was performed with a Philips Gemini TF 16 scanner with lutetium-yttrium oxyorthosilicate (LYSO; lutetium-to-yttrium ratio, 9:1) scintillation crystals, time of flight (TOF) capability (Philips Astonish TF technology) and a reported system sensitivity of 6.6 cps/kBq [13]. Patients had to fast for ≥6h prior to the [$^{18}$F]FDG injection. A median activity of 267 MBq [$^{18}$F]FDG (IQR, 246 to 326 MBq; median, 3.8 MBq/kg; IQR, 3.1 to 4.6 MBq/kg) was administered intravenously. PET scan was performed after a median uptake time of 70 minutes (IQR, 62 to 85 min) resulting in a decay corrected activity of 2.3 MBq/kg at imaging start (IQR, 2.0 to 2.9 MBq/kg). PET data was acquired from base of skull to the proximal femora in 3D acquisition mode (transaxial field of view, 71.6 cm; acquisition time, 1.5 to 3 minutes per bed position; bed overlap, 53.3%; median product of uncorrected injected activity per kg and acquisition time [AT], 7.8 (MBq/kg)*min; IQR, 6.6 to 9.2 (MBq/kg)*min). In 283 patients, a non-enhanced CT (usually low-dose) was used for attenuation correction (automated tube current modulation; maximum tube current-time product, 50 to 200 mAs; tube voltage, 120 kV; gantry rotation time, 0.5 s). In 102 patients, attenuation correction used a contrast-enhanced diagnostic CT (automated tube current modulation; maximum tube current-time product, 200 mAs; tube voltage, 120 kV; delay after contrast agent injection, 80 seconds; bolus rate, 3 mL/s).

In the validation cohort, [$^{18}$F]FDG-PET/CT imaging was performed with a GE Discovery MI scanner with silicon photomultipliers (SiPM) in a 3-ring detector setting, TOF capability and a reported system sensitivity of 7.3 cps/kBq [14]. A median activity of 261 MBq [$^{18}$F]FDG (IQR, 248 to 301 MBq; median, 3.8 MBq/kg; IQR, 3.3 to 4.3 MBq/kg) was administered intravenously. Median uptake time was 65 minutes (IQR, 60 to 73 min), and decay corrected activity at imaging start was 2.5 MBq/kg (IQR, 2.1 to 2.9 MBq/kg). PET data was acquired from base of

skull to the proximal femora (3D mode; transaxial field of view, 70 cm; acquisition time, 2 to 3 minutes per bed position; bed overlap, approx. 25%; median AT, 9.3 (MBq/kg)*min; IQR, 8.0 to 11.6 (MBq/kg)*min). A non-enhanced CT (usually low-dose) was used for attenuation correction (automated tube current modulation “Smart mA”; maximum tube current-time product, 100 to 200 mAs; tube voltage, 120 kV; gantry rotation time, 0.5 s).

**[$^{18}$F]FDG-PET/CT image reconstruction**

PET raw data in the training+test cohort were reconstructed using iterative reconstruction (ordered subset expectation maximization; OSEM) with TOF analysis (BLOB-OS-TF; iterations, 3; subsets, 33; filter, ‘smooth’ [kernel width, 14.1 cm; relaxation parameter, 0.7]; matrix, 144 × 144; voxel size, 4.0 × 4.0 × 4.0 mm$^3$). CT raw data were reconstructed with a slice thickness of 5 millimeters for attenuation correction and with a slice thickness of 3 mm for visual assessment (convolution kernel, B (soft tissue)).

In the validation cohort, PET raw data were reconstructed iteratively with Bayesian penalized likelihood reconstruction (GE “Q.Clear”) with a penalization factor β of 450 (matrix, 256 × 256; voxel size, 2.73 × 2.73 × 2.78 mm$^3$) [15]. Non-enhanced CT raw data were reconstructed with a slice thickness of 3.75 millimeters for attenuation correction (convolution kernel, Q AC) and with a slice thickness of 1.25 mm for visual assessment (convolution kernel, standard (soft tissue)).

**PET: SUV measurements**

<u>Background SUVmean</u>

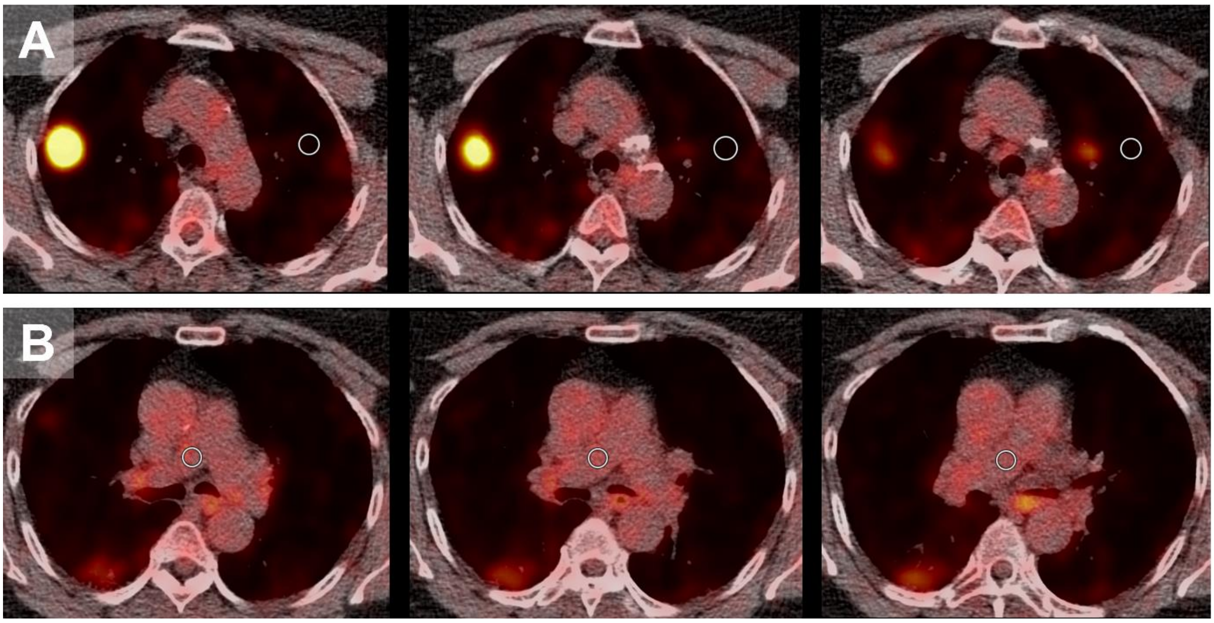


**Figure S2.1:** Case example of SUVmean measurements for lung background (A) and mediastinum background (B) in a patient with a primary tumor in the right upper lobe.

Primary tumor SUVmax

Partial volume correction of the primary tumor SUVmax was performed based on the method described by Vesselle *et al.* (2000) using a size-dependent recovery coefficient (RC) [17]:

$$PV\ corrected\ SUVmax = lung\ background\ SUVmean + \frac{primary\ tumor\ SUVmax - lung\ background\ SUVmean}{RC}$$

RCs for the SUVmax were determined for each PET/CT scanner from phantom measurements with an IEC NEMA Body phantom equipped with six “hot” spheres with diameters of 10, 13, 17, 22, 28, and 37 mm, respectively. Based on these results, the following RCs were applied to primary tumors with different diameters:

| Primary tumor size (mm) | RC (training+test cohort) | RC (validation cohort) |
|---|---|---|
| ≤11.5 | 0.5 | 1.08 |
| >11.5-15.0 | 0.91 | 1.15 |
| >15.0-19.5 | 1.2 | 1.11 |
| >19.5-25 | 1.29 | 1.12 |
| >25 | 1.24 | 1.15 |

**PET: Retrospective smoothing of PET data in the validation cohort**

Reconstructed spatial resolution

Reconstructed spatial resolution for both PET scanners with the image reconstruction protocols specified above were obtained using a NEMA IEC phantom equipped with six spheres filled with [$^{18}$F]FDG in a sphere-to-background ratio of 8:1 [15]. Reconstructed spatial resolution was calculated by modeling the point spread function (PSF) of the sphere inserts in the reconstructed phantom images by a 3D Gaussian [18].

The original PET data of the validation cohort scanner had a reconstructed spatial resolution of 4.7 mm full width at half maximum (FWHM), as reported before [15]. PET data from the training+test cohort scanner showed a spatial resolution of 7.8 mm FWHM (*not published before*).

Smoothing of PET data

To harmonize the reconstructed spatial resolution of both PET scanners, the reconstructed PET data acquired with the validation cohort scanner were retrospectively smoothed with a Gaussian filter of 6.2 mm FWHM according to the formula [24]:

$$FWHM_{target}{}^2 = FWHM_{original}{}^2 + FWHM_{filter}{}^2 \qquad (1)$$

with a target resolution of 7.8 mm FWHM (training+test cohort scanner) and an original spatial resolution of 4.7 mm FWHM (validation cohort scanner).
