# Supplementary material for: A machine learning tool to improve prediction of mediastinal lymph node metastases in non-small cell lung cancer using routinely obtainable [18F]FDG-PET/CT parameters
Source: Eur J Nucl Med Mol Imaging. 2023 Feb 23;50(7):2140–51. doi: 10.1007/s00259-023-06145-z (PMC10199849; doi:10.1007/s00259-023-06145-z)
Supplement: Supplementary file 3 — Supplementary file3 (PDF 338 KB) [file 259_2023_6145_MOESM3_ESM.pdf]

Supplementary material #3

Article title: A machine learning tool to improve prediction of mediastinal lymph node metastases in non-small cell lung cancer using routinely obtainable [^{18}F]FDG-PET/CT parameters

Journal name: European Journal of Nuclear Medicine and Molecular Imaging

Author names: Julian M.M. Rogasch, Liza Michaels, Georg L. Baumgärtner, Nikolaj Frost, Jens-Carsten Rückert, Jens Neudecker, Sebastian Ochsenreither, Manuela Gerhold, Bernd Schmidt, Paul Schneider, Holger Amthauer, Christian Furth, Tobias Penzkofer

Corresponding author:

Julian M.M. Rogasch
Department of Nuclear Medicine
Charité – Universitätsmedizin Berlin
Augustenburger Platz 1
D-13353 Berlin, Germany
Phone: +49 30 450 627106
Fax: +49 30 450 7557338
e-mail: julian.rogasch@charite.de

This Supplementary material #3 contains details on the investigated features and the machine learning methodology. The python and R scripts can be found in the Open Data repository zenodo (https://doi.org/10.5281/zenodo.7094287). It also contains a dictionary that explains how each of the variables and its categories were defined.

**Feature**	**Variable type**
[^{18}F]FDG-PET/CT (n=23)	
Toney *et al.*: Primary tumor SUVmax (PV-corrected)	continuous
Toney *et al.*: Primary tumor diameter [mm]	continuous
Toney *et al.*: N1 LN SUVmax (BG-corrected)	continuous
Toney *et al.*: N2 LN SUVmax (BG-corrected)	continuous
Toney *et al.*: N3 LN SUVmax (BG-corrected)	continuous
Toney *et al.*: N1 LN short axis diameter [mm]	continuous
Toney *et al.*: N2 LN short axis diameter [mm]	continuous
Toney *et al.*: N3 LN short axis diameter [mm]	continuous
Primary tumor SUVmax (non-corrected)	continuous
N1 LN SUVmax (non-corrected)	continuous
N2 LN SUVmax (non-corrected)	continuous
N3 LN SUVmax (non-corrected)	continuous
Visual PET score (ipsilateral hilar LNs)	categorical
Visual PET score (mediastinal LNs)	categorical
Visual PET score (contralateral hilar LNs)	categorical
N1 LN CT-positive (i.e., size >10 mm or p.c. infiltrated)	binary
N2/3 LN CT-positive (i.e., size >10 mm or p.c. infiltrated)	binary
N1 LN PET-positive (visual score ≥ 3)	binary
N2/3 LN PET-positive (visual score ≥ 3)	binary
N1 LN PET/CT-positive (i.e., CT- and/or PET-positive with score ≥ 3)	binary
N2/3 LN PET/CT-positive (i.e., CT- and/or PET-positive with score ≥ 3)	binary
N1 LN PET/CT-positive (CT-positive or PET-positive with score ≥ 2)	binary
N2/3 LN PET/CT-positive (CT-positive or PET-positive with score ≥ 2)	binary
Clinical and pathological data (n=17)	
Age [years]	continuous
Sex	categorical
NSCLC subtype	categorical
Histological grade of differentiation (G)	categorical
c/pT stage	categorical
Primary tumor side	categorical

Primary tumor lobe	categorical
ESTS/ESMO guidelines: Primary tumor peripheral vs. central	binary
ESTS/ESMO guidelines: Primary tumor diameter >3 cm	binary
ESTS/ESMO guidelines: Sum of risk factors for unexpected pN2 (N1 LNs rated PET-positive at score ≥ 3)	categorical
ESTS/ESMO guidelines: Sum of risk factors for unexpected pN2 (N1 LNs rated PET-positive at score ≥ 2)	categorical
Smoking behavior (never vs. former vs. current)	categorical
Pack years	continuous
Work-related exposure to inhalable toxins	binary
Acute inflammatory pulmonary disease	binary
Structural pulmonary disease (different categories)	categorical
Anti-inflammatory or immunosuppressive medication or immunosuppressive disease (different categories)	categorical

Table S3.1. Full list of 40 features before one-hot encoding.

PV, partial volume; LN, lymph node; BG, background; p.c., *per continuum*; ESTS, European Society of Thoracic Surgeons; ESMO, European Society for Medical Oncology

**Feature**	**Encoding**
[18F]FDG-PET/CT (n=32)	
Toney *et al.*: Primary tumor SUVmax (PV-corrected)	*continuous*
Toney *et al.*: Primary tumor diameter [mm]	*continuous*
Toney *et al.*: N1 LN SUVmax (BG-corrected)	*continuous*
Toney *et al.*: N2 LN SUVmax (BG-corrected)	*continuous*
Toney *et al.*: N3 LN SUVmax (BG-corrected)	*continuous*
Toney *et al.*: N1 LN short axis diameter [mm]	*continuous*
Toney *et al.*: N2 LN short axis diameter [mm]	*continuous*
Toney *et al.*: N3 LN short axis diameter [mm]	*continuous*
Primary tumor SUVmax (non-corrected)	*continuous*
N1 SUVmax (non-corrected)	*continuous*
N2 SUVmax (non-corrected)	*continuous*
N3 SUVmax (non-corrected)	*continuous*
Visual PET score (ipsilateral hilar LNs)	1, 2, 3, 4
Visual PET score (mediastinal LNs)	1, 2, 3, 4
Visual PET score (contralateral hilar LNs)	1, 2, 3, 4
N1 LN CT-positive (i.e., size >10 mm or p.c. infiltrated)	*binary*
N2/3 LN CT-positive (i.e., size >10 mm or p.c. infiltrated)	*binary*
N1 LN PET-positive (visual score ≥ 3)	*binary*
N2/3 LN PET-positive (visual score ≥ 3)	*binary*
N1 LN PET/CT-positive (i.e., CT- and/or PET-positive with score ≥ 3)	*binary*
N2/3 LN PET/CT-positive (i.e., CT- and/or PET-positive with score ≥ 3)	*binary*
N1 LN PET/CT-positive (CT-positive or PET-positive with score ≥ 2)	*binary*
N2/3 LN PET/CT-positive (CT-positive or PET-positive with score ≥ 2)	*binary*
Clinical and pathological data (n=48)	
Age [years]	*continuous*
Sex	1, 2
NSCLC subtype	1, 2, 3
Histological grade of differentiation (G)	1, 2, 3, 4
c/pT stage	11, 12, 13, 21, 22, 30, 40
Primary tumor side	1, 2
Primary tumor lobe	1, 2, 3, 8
ESTS/ESMO guidelines: Primary tumor peripheral vs. central	*binary*
ESTS/ESMO guidelines: Primary tumor diameter >3 cm	*binary*
ESTS/ESMO guidelines: Sum of risk factors for unexpected pN2 (N1 LNs rated PET-positive at score ≥ 3)	0, 1, 2, 3
ESTS/ESMO guidelines: Sum of risk factors for unexpected pN2 (N1 LNs rated PET-positive at score ≥ 2)	0, 1, 2, 3

Smoking behavior (never vs. former vs. current)	0, 1, 2
Pack years	*continuous*
Work-related exposure to inhalable toxins	*binary*
Acute inflammatory pulmonary disease	*binary*
Structural pulmonary disease (different categories)	0, 1, 2, 3, 4
Anti-inflammatory or immunosuppressive medication or immunosuppressive disease (different categories)	0, 1, 2, 3

Table S3.2. Full list of 80 features after one-hot encoding.

This is the final list of features used for machine learning. In categorical variables, each of the listed categories represents one dummy variable after one-hot encoding. Binary variables were not subjected to one-hot encoding.

PV, partial volume; LN, lymph node; BG, background; p.c., *per continuum*; ESTS, European Society of Thoracic Surgeons; ESMO, European Society for Medical Oncology

**Feature**	**Variable type**
Toney *et al.*: Primary tumor SUVmax (PV-corrected)	continuous
Toney *et al.*: Primary tumor diameter [mm]	continuous
Toney *et al.*: N1 LN SUVmax (BG-corrected)	continuous
Toney *et al.*: N2 LN SUVmax (BG-corrected)	continuous
Toney *et al.*: N3 LN SUVmax (BG-corrected)	continuous
Toney *et al.*: N1 LN short axis diameter [mm]	continuous
Toney *et al.*: N2 LN short axis diameter [mm]	continuous
Toney *et al.*: N3 LN short axis diameter [mm]	continuous

Table S3.3. Full list of 8 features according to Toney et al.
This is the final list of features used for the artificial neural network based on the publication by Toney *et al.* [8]. These 8 features were also included in the other machine learning models (see Table S3.1 and S3.2).
PV, partial volume; LN, lymph node; BG, background

Machine learning

Feature preprocessing: Imputation

Variables with missing data included the NSCLC subtype (4/491 patients), smoking behavior (2/491), pack years (12/491), and work-related exposure to inhalable toxins (3/491). In 105/491 patients that did not undergo primary tumor surgery, the histological grade of differentiation was unknown. In 2 patients, the T stage could not be determined. These missing data were imputed with multivariate imputation by chained equations (MICE) using the *mice* package in R (R version 4.1.3; The R Project for Statistical Computing).

Feature preprocessing: One-hot encoding

Categorical variables were subjected to one-hot encoding (*get_dummies* function in the pandas package for python 3). Binary variables with categories (0, 1) remained unchanged.

Feature preprocessing: Robust scaling

Due to the presence of outliers in several continuous variables, all variables were scaled with the *RobustScaler* in the sklearn module for python (python version 3.9). This was performed as part of a *pipeline* to ensure that no data leakage could occur during training.

	**Hyperparameter**	**Parameter space**
RF	n_estimators<br>max_depth<br>max_features	10, 50, 100, 200, 500<br>None, 1, 2, 3, 4, 5, 6<br>None, “sqrt”, “log2”
SVC	C<br>kernel<br>degree<br>gamma<br>class_weight	0.1, 0.25, 0.5, 0.75, 1.0, 2.5, 5.0<br>“rbf”, “linear”, “sigmoid”<br>1, 2, 3, 4, 5, 6, 7, 8, 9, 10<br>“scale”, “auto”<br>None, “balanced”
GBM	learning_rate<br>n_estimators<br>subsample<br>max_depth	6 values in a logarithmic space from 0.0001 to 1<br>10, 50, 100, 200, **500**<br>0.1, **0.2**, 0.4, 0.6, 0.8, 1.0<br>**1**, 2, 3, 4, 5, 6
XGB	learning_rate<br>subsample<br>n_estimators<br>max_depth<br>booster"	6 values in a logarithmic space from 0.0001 to 1<br>0.1, 0.2, 0.4, 0.6, 0.8, 1.0<br>10, 50, 100, 200, 500<br>1, 2, 3, 4, 5, 6<br>"gbtree", "gblinear", "dart"
MLP	hidden_layer_sizes<br>solver<br>activation<br>alpha	(n/2),(n/2,n/2,n/2),(n/2,n/4,n/4),(n/2,n/4,n/8),(n/2,n/2,n/4,n/4,n/8)<br>"lbfgs", "sgd", "adam"<br>"identity", "logistic", "tanh", "relu"<br>0.00001, 0.0001, 0.001, 0.01, 0.1

Table S3.4. All hyperparameters that were investigated during grid search.

Best parameters for the gradient boosting classifier (GBM) are highlighted in bold (best learning_rate: 0.00398).

RF, random forest; SVC, support vector machine; XGB, XGBoost; MLP, multi-layer perceptron
