# Supplementary material for: A machine learning tool to improve prediction of mediastinal lymph node metastases in non-small cell lung cancer using routinely obtainable [18F]FDG-PET/CT parameters
Source: Eur J Nucl Med Mol Imaging. 2023 Feb 23;50(7):2140–51. doi: 10.1007/s00259-023-06145-z (PMC10199849; doi:10.1007/s00259-023-06145-z)
Supplement: Supplementary file 4 — Supplementary file4 (PDF 990 KB) [file 259_2023_6145_MOESM4_ESM.pdf]

Supplementary material #4

Article title: A machine learning tool to improve prediction of mediastinal lymph node metastases in non-small cell lung cancer using routinely obtainable [^{18}F]FDG-PET/CT parameters

Journal name: European Journal of Nuclear Medicine and Molecular Imaging

Author names: Julian M.M. Rogasch, Liza Michaels, Georg L. Baumgärtner, Nikolaj Frost, Jens-Carsten Rückert, Jens Neudecker, Sebastian Ochsenreither, Manuela Gerhold, Bernd Schmidt, Paul Schneider, Holger Amthauer, Christian Furth, Tobias Penzkofer

Corresponding author:

Julian M.M. Rogasch
Department of Nuclear Medicine
Charité – Universitätsmedizin Berlin
Augustenburger Platz 1
D-13353 Berlin, Germany
Phone: +49 30 450 627106
Fax: +49 30 450 7557338
e-mail: julian.rogasch@charite.de

This Supplementary material #4 contains details on the diagnostic performance of visual PET/CT reading and model performance results of all machine learning models. Furthermore, detailed results for visual PET scores and SUVmax in both cohorts, including the impact of retrospectively smoothing the PET data, are shown. Case examples illustrate discordant findings of the visual assessment vs. the GBM.

**Model**	**AUC**	**Sensitivity**	**Specificity**	**Accuracy**
Visual assessment: Only mediastinal LNs decisive				
Visual PET score	0.87 (0.83-0.91)	---	---	---
≥2	---	0.89 (0.82-0.93)	0.59 (0.53-0.65)	0.7 (0.65-0.74)
≥3	---	0.86 (0.79-0.91)	0.72 (0.66-0.77)	0.77 (0.72-0.81)
PET score ≥2 and/ or LN size >10 mm	---	0.92 (0.86-0.96)	0.53 (0.47-0.6)	0.68 (0.63-0.72)
Visual assessment: Mediastinal LNs and/or contralateral hilar LNs decisive				
Visual PET score	0.86 (0.82-0.9)	---	---	---
≥2	---	0.92 (0.86-0.96)	0.38 (0.32-0.45)	0.58 (0.53-0.63)
≥3	---	0.87 (0.8-0.92	0.64 (0.57-0.7)	0.72 (0.67-0.76)
PET score ≥2 and/ or LN size >10 mm	---	0.95 (0.9-0.98)	0.35 (0.29-0.42)	0.57 (0.52-0.62)

Table S4.1. Diagnostic performance of visual PET/CT reading in the training+test cohort. Results in parentheses are 95% confidence intervals. PET score ≥2 means that uptake of the lymph node (LN) is higher than the mediastinal blood pool. PET score ≥3 means uptake at least as high as the normal liver uptake.

If the assessment of pathological LNs was not limited to mediastinal LNs but extended to contralateral hilar LNs, specificity reduced (because isolated contralateral hilar LN uptake is often non-malignant) but did not benefit the sensitivity.

Model	AUC	Sensitivity	Specificity	Accuracy
Visual assessment: Only mediastinal LNs decisive				
Visual PET score	0.91 (0.85-0.97)	---	---	---
≥2	---	0.96 (0.86-1.0)	0.58 (0.44-0.71)	0.75 (0.66-0.83)
≥3	---	0.96 (0.86-1.0)	0.7 (0.57-0.82)	0.82 (0.73-0.89)
PET score ≥2 and/ or LN size >10 mm	---	0.96 (0.86-1.0)	0.58 (0.44-0.71)	0.75 (0.66-0.83)
Visual assessment: Mediastinal LNs and/or contralateral hilar LNs decisive				
Visual PET score	0.91 (0.85-0.97)	---	---	---
≥2	---	0.98 (0.89-1.0)	0.4 (0.28-0.54)	0.67 (0.57-0.76)
≥3	---	0.96 (0.86-1.0)	0.58 (0.44-0.71)	0.75 (0.66-0.83)
PET score ≥2 and/ or LN size >10 mm	---	0.98 (0.89-1.0)	0.4 (0.28-0.54)	0.67 (0.57-0.76)

Table S4.2. Diagnostic performance of visual PET/CT reading in the validation cohort. Results in parentheses are 95% confidence intervals. PET score ≥2 means that uptake of the LN is higher than the mediastinal blood pool.

Model	n features	Feature selection	Test AUC	Test SD	Validation AUC
RF	30	GBM	.918	.047	.929
RF	25	GBM	.918	.047	.929
RF	20	GBM	.918	.047	.929
RF	15	GBM	.912	.043	.929
RF	10	GBM	.907	.051	.930
RF	5	GBM	.910	.058	.917
RF	1	MIC	.864	.065	.934
SVC	30	GBM	.912	.046	.932
SVC	25	GBM	.912	.046	.932
SVC	20	GBM	.912	.046	.932
SVC	15	RF	.916	.048	.935
SVC	10	RF	.912	.047	.932
SVC	5	GBM	.911	.043	.916
SVC	1	RF	.876	.062	.903
GBM	30	GBM	.917	.047	.939
GBM	25	GBM	.917	.046	.939
GBM	20	AdaBoost	.919	.050	.938
GBM	15	RF	.920	.048	.938
**GBM**	**10**	**GBM**	**.914**	**.050**	**.941**
GBM	5	GBM	.910	.052	.925
GBM	1	RF	.868	.065	.926
XGB	30	GBM	.914	.053	.933
XGB	25	GBM	.914	.053	.933
XGB	20	AdaBoost	.915	.048	.931
XGB	15	GBM	.914	.053	.933
XGB	10	AdaBoost	.911	.052	.925
XGB	5	RF	.903	.050	.926
XGB	1	RF	.867	.074	.932
MLP	30	GBM	.913	.055	.925
MLP	25	GBM	.908	.064	.926
MLP	20	GBM	.918	.050	.933
MLP	15	RF	.905	.055	.927
MLP	10	GBM	.897	.059	.924
MLP	5	RF	.889	.075	.948
MLP	1	MIC	.881	.068	.925

Table S4.3. Diagnostic performance of various models and n features.

This table lists for each model the feature selection method that achieved the highest test AUC with a predefined number of features in the model. Test AUC is the mean test AUC (with its standard deviation [SD]) of the 10 folds during cross-validation. The final model is highlighted in bold. It was chosen based on high test AUC at a low number of features in the model.

RF, random forest; GBM, gradient boosting; MIC, mutual information classifier; SVC, support vector classifier; XGB, XGBoost; MLP, multi-layer perceptron

**Patients with N0/1**										
Training+test cohort						*Validation cohort*				
		**Visual assessment**						**Visual assessment**		
		FP	TN					FP	TN	
**GBM**	FP	64	**8**	*72*		**GBM**	FP	9	**1**	*10*
	TN	50	122	*172*			TN	15	32	*47*
		114	*130*	*244*				*24*	*33*	*57*

**Patients with N2/3**										
Training+test cohort						*Validation cohort*				
		**Visual assessment**						**Visual assessment**		
		FN	TP					FN	TP	
**GBM**	FN	8	**6**	*14*		**GBM**	FN	1	**3**	*4*
	TP	3	124	*127*			TP	1	44	*45*
		11	*130*	*141*				*2*	*47*	*49*

Table S4.4. Cross tables with the results of visual assessment and the machine learning classifier (GBM)

Visual assessment used a PET score ≥2 and/ or LN size >10 mm, and the GBM was rated positive at a probability threshold of >0.19. Cases that were classified incorrectly by the GBM but correctly with visual assessment are highlighted in bold.

FP, false positive; TN, true negative; FN, false negative; TP, true positive

SUVmax	N0/1		p	N2/3		p
	Training+test	*Validation*		*Training+test*	*Validation*	
N1	2.5 (0.7-3.8)	2.9 (0.6-3.7)	0.59	8.0 (4.0-13.4)	11.5 (6.4-14.5)	**0.02**
N2	1.9 (1.5-3.0)	1.9 (1.5-3.1)	0.55	8.2 (4.5-13.8)	10.2 (6.6-14.3)	0.11
N3	2.1 (1.5-2.8)	2.2 (1.5-3.1)	0.37	2.8 (1.7-4.5)	3.3 (2.4-6.0)	0.14
N1 (BG)	4.9 (0.7-8.4)	7.4 (0.6-10.6)	0.1	15.4 (7.7-25.3)	22.9 (14.0-42.0)	**<0.001**
N2 (BG)	1.7 (1.4-2.2)	1.7 (1.5-2.0)	0.82	5.5 (2.9-7.8)	5.9 (3.9-9.8)	0.09
N3 (BG)	1.7 (1.4-1.9)	1.7 (1.5-2.0)	0.49	1.8 (1.5-3.3)	2.1 (1.7-3.4)	0.22
Primary	11.6 (6.4-16.1)	10.6 (7.7-15.7)	0.96	13.8 (9.5-18.0)	14.9 (9.2-18.3)	0.45
Primary (PVC)	9.6 (5.6-13.2)	9.5 (6.9-13.7)	0.6	11.6 (8.3-14.7)	13.0 (8.0-15.9)	0.13

Table S4.5. SUVmax separated by the two cohorts (PET scanners)

Median with IQR of SUVmax from lymph node regions and primary tumors are displayed. Differences in median SUVmax between the two cohorts (original PET data) were non-significant except for N1 (non-corrected) and N1 lymph nodes with background correction (Wilcoxon rank-sum test).

BG, background corrected; PVC, partial volume corrected

Score	N0/1		p-value	N2/3		p-value
	Training+test	*Validation*		*Training+test*	*Validation*	
1	74	15	0.1	10	0	0.13
2	67	11		9	1	
3	58	23		20	6	
4	45	8		102	42	

Table S4.6. Visual PET scores for ipsilateral hilar lymph nodes

Differences in the distribution of visual PET scores between the two cohorts (i.e., PET scanners) were not significant (Fisher's exact test).

Score	N0/1		p-value	N2/3		p-value
	Training+test	*Validation*		*Training+test*	*Validation*	
1	144	33	0.67	16	2	0.25
2	31	7		4	0	
3	54	11		13	3	
4	15	6		108	44	

Table S4.7. Visual PET scores for mediastinal lymph nodes

Differences in the distribution of visual PET scores between the two cohorts (i.e., PET scanners) were not significant (Fisher's exact test).

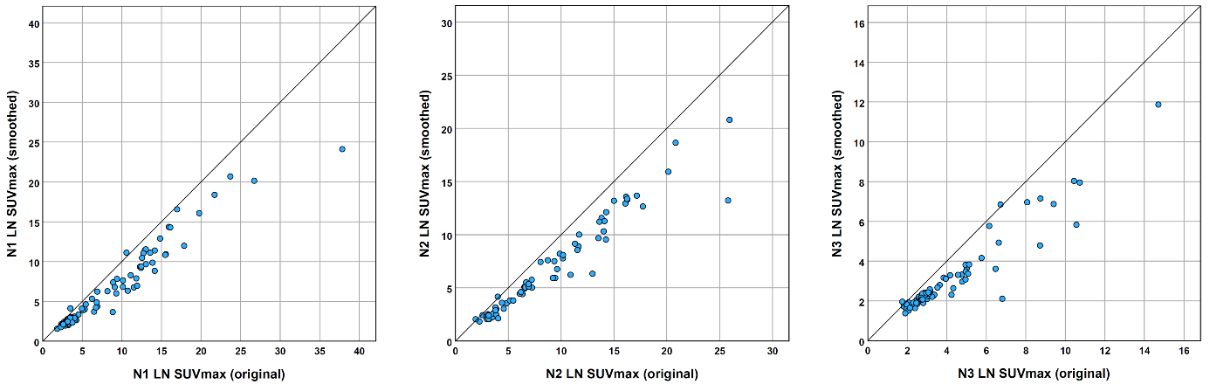

Figure S4.8. Lymph node SUVmax: Original vs. smoothed PET data (validation cohort)
In the validation cohort, the impact of retrospectively smoothing the original PET data on lymph node SUVmax was evaluated. SUVmax were systematically and significantly lower in the smoothed PET data. The effect was similar for the N1 vs. N2 vs. N3 region. The black line is the reference line.

		**Smoothed**				
		1	2	3	4	
**Original**	1	35	-	-	-	*35*
	2	**3**	4	-	-	*7*
	3	-	**5**	9	-	*14*
	4	-	-	**4**	46	*50*
		38	*9*	*13*	*46*	*106*

Table S4.9. Visual PET scores for mediastinal lymph nodes: Original vs. smoothed data
In the validation cohort, the impact of retrospectively smoothing the original PET data on visual PET scores was evaluated. Scores for the mediastinal lymph nodes were lower in 12 of the 106 patients (highlighted in bold).

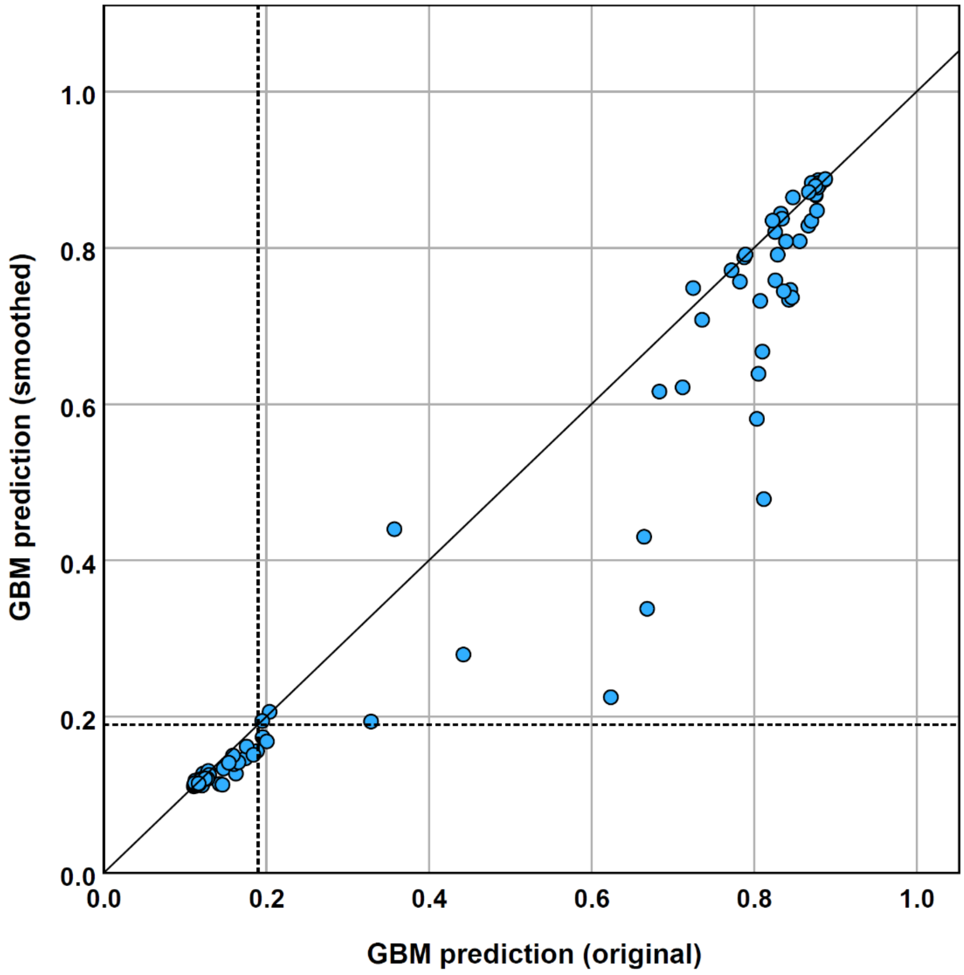

Figure S4.10. Predicted probability of the GBM model: Original vs. smoothed data

In the validation cohort, the impact of retrospectively smoothing the original PET data and thereby decreasing SUVmax and visual PET scores was evaluated with respect to performance of the GBM model. Predicted probabilities of the model were, on average, lower with the smoothed PET data (the black line is the reference line). However, if the threshold of a probability >0.19 was used to distinguish a positive result of the model (dashed lines), only two patients were classified discordantly compared to the original data.

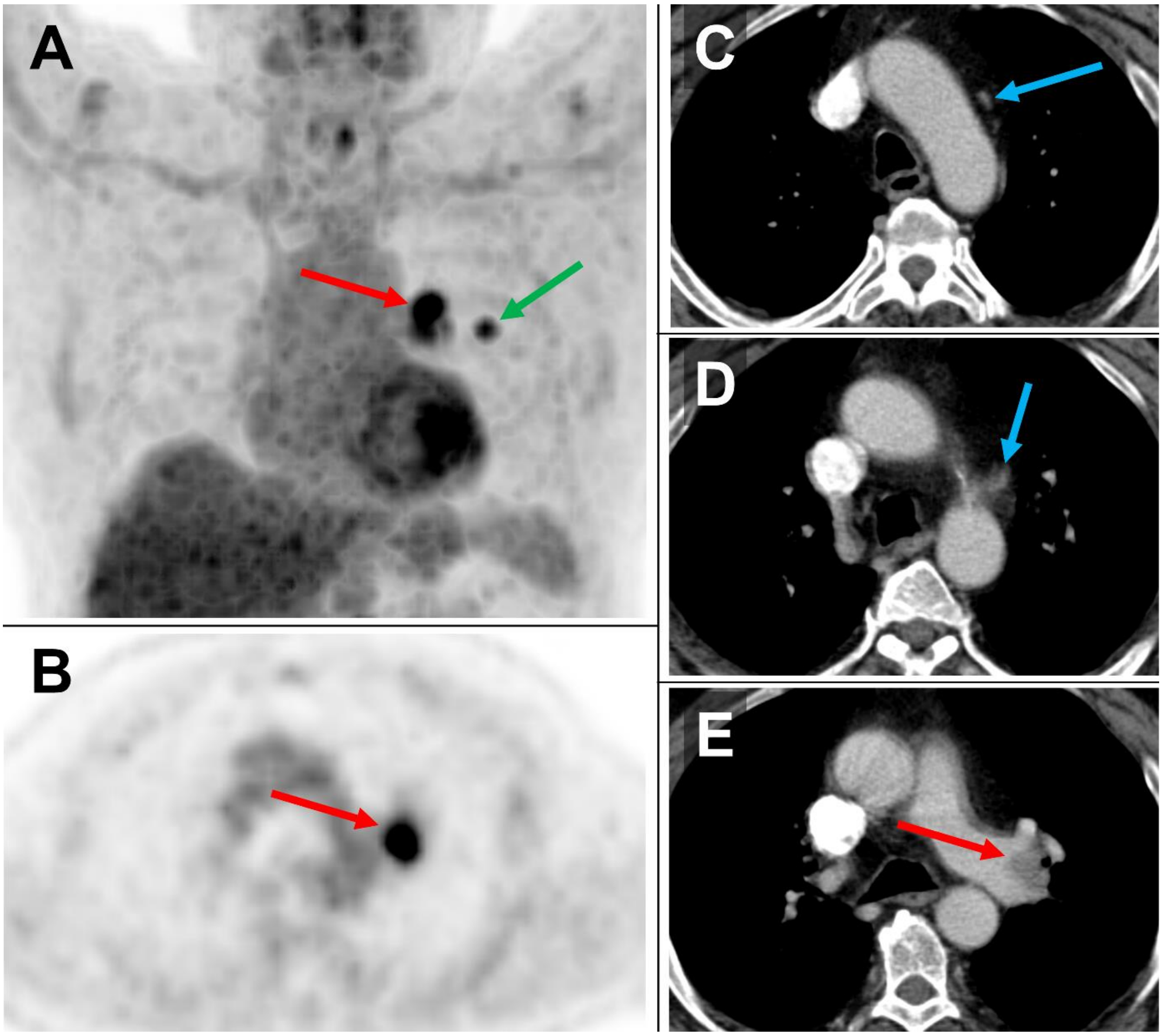

Figure S4.11. Case example #2: GBM true positive

63-year-old male with a G3 adenocarcinoma (20 mm) in the left upper lobe (A, green arrow). This patient was part of the training+test cohort. FDG-PET/CT also revealed a large (short axis, 14 mm) and intensely FDG-avid N1 lymph node (visual PET score = 4; A+B+E, red arrow). However, all N2 and N3 lymph nodes were unremarkable both in FDG-PET (score = 1) and CT (short axis < 10 mm), including paraaortal and subaortic lymph nodes (C+D, blue arrow). The patient underwent surgery with systematic lymph node dissection, which revealed unsuspected pN2 in the paraaortal and subaortic lymph nodes. Predicted probability of the GBM was 0.193 and therefore truly positive for N2/3 (threshold, >0.19), probably due to the highly suspicious N1 lymph node, which increases the predicted probability of N2/3. This result was discordant to the false negative visual assessment.

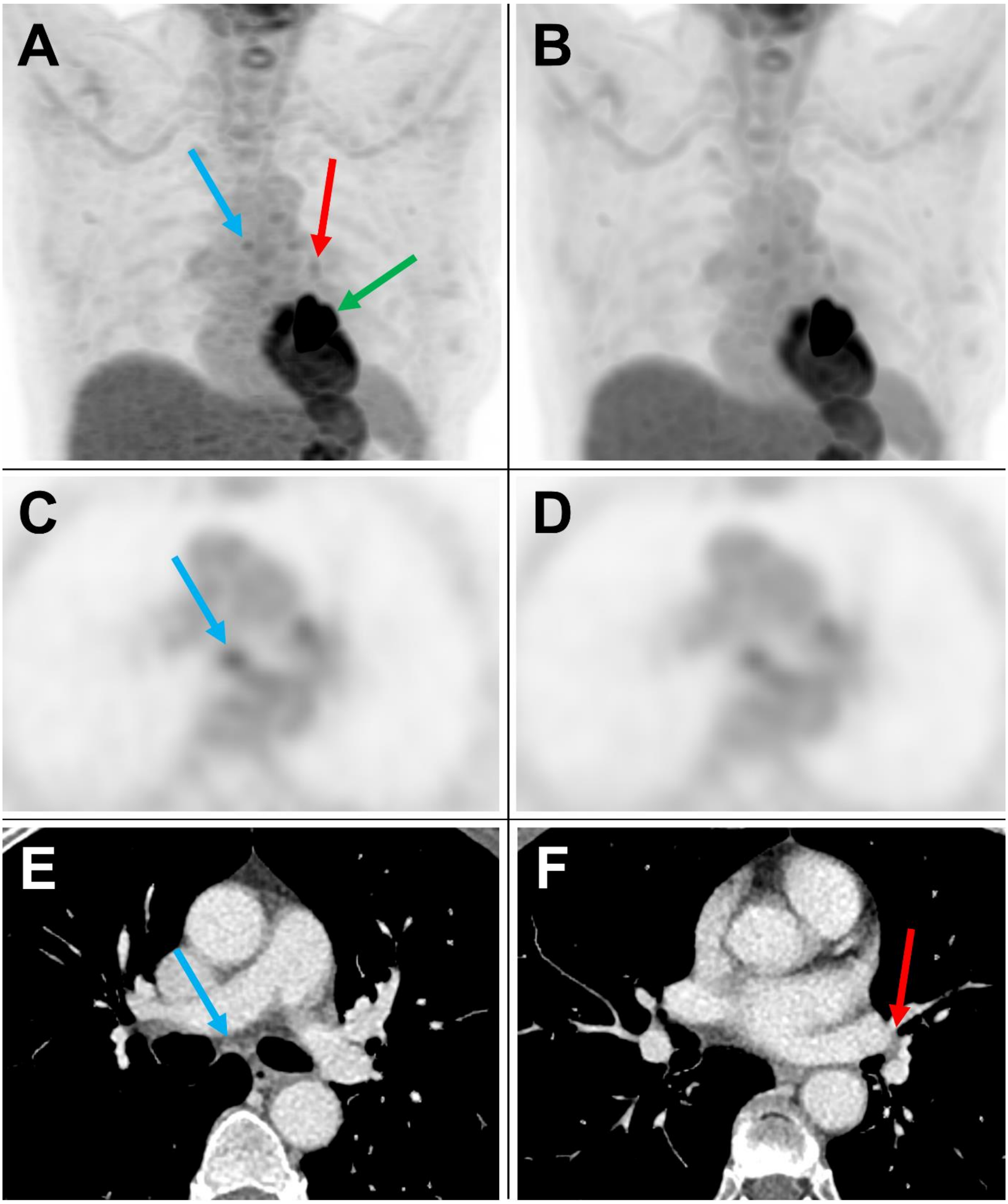

Figure S4.12. Case example #3: GBM false negative

63-year-old female from the validation cohort with a G3 adenocarcinoma of the left lower lobe (36 mm). FDG-PET/CT showed the intensely FDG-positive retrocardiac primary tumor (green arrow). Furthermore, a subcarinal lymph node showed moderately increased FDG uptake (similar to the normal liver = visual score 3) but measured only 4 mm in short axis. Based on the FDG-PET finding, visual assessment was positive for N2/3. The N1 lymph nodes (red arrow) showed slightly increased uptake (visual score = 2) and were also small.

Surgery revealed pN2 in two subcarinal lymph nodes. The GBM was false negative (predicted probability: 0.12). This was likely because all lymph nodes, including the N1 station, were small and showed comparably low SUVmax (N2: 2.6; N1: 2.4), rendering most features in the model negative.

Figure B and D show the retrospectively smoothed PET data. The lower spatial resolution can be appreciated visually, and the visual PET score of the subcarinal lymph node would be 2 (instead of 3 in the original data). SUVmax were 2.4 (N2) and 2.2 (N1). Predicted probability of the GBM was 0.12 with the smoothed PET data, unchanged compared to the original data.

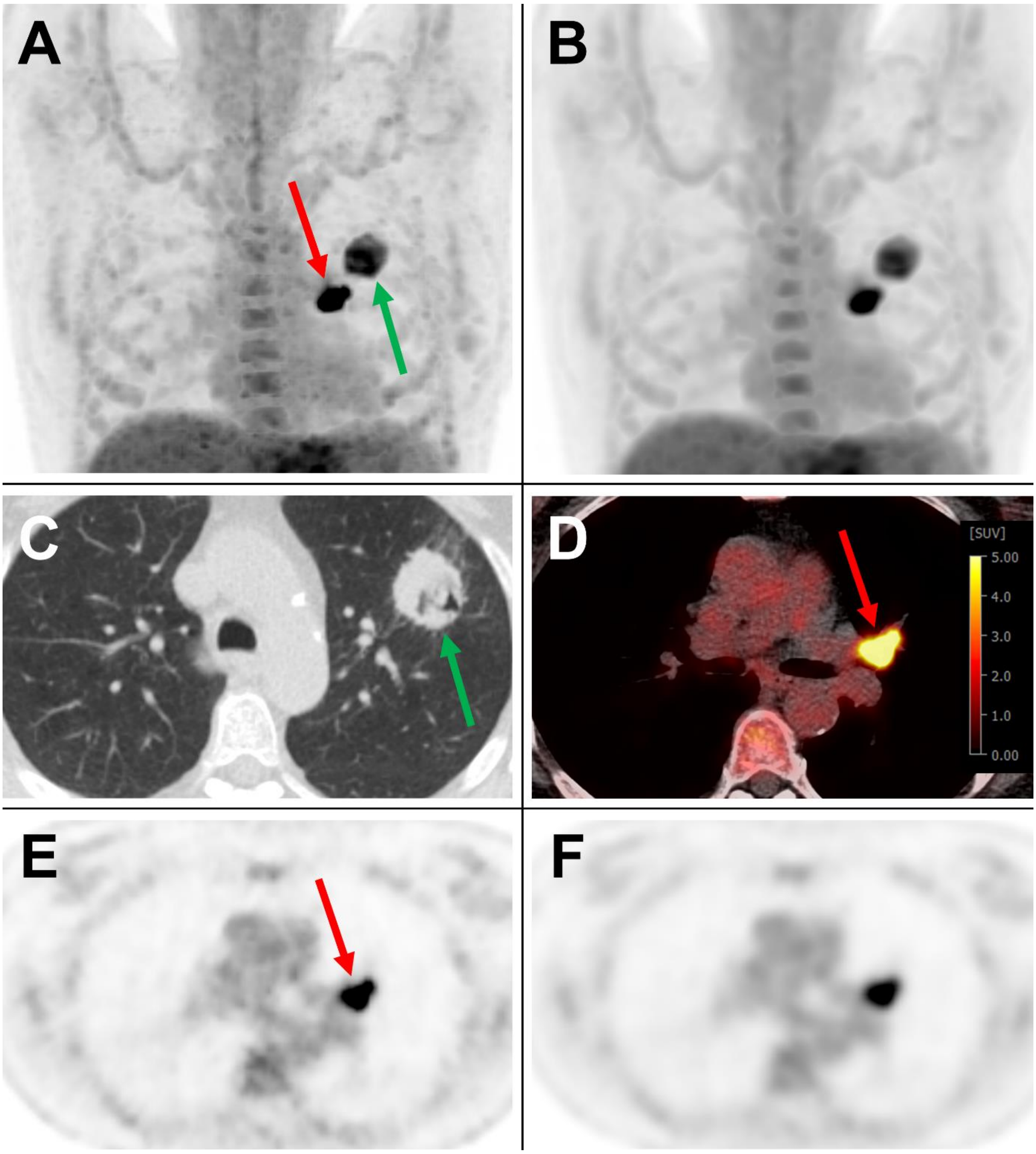

Figure S4.13. Case example #4: GBM false positive

66-year-old female from the validation cohort with a G2 squamous cell carcinoma of the left upper lobe (35 mm; green arrows). The N1 lymph node station (red arrows) was positive both in FDG-PET (SUVmax 13.9; visual score = 4) and CT (short axis, 13 mm). However, N2 lymph nodes were negative by visual assessment. Surgery confirmed pN0. Predicted probability of the GBM was 0.20, i.e. false positive for N2/3 (>0.19), likely due to the highly suspicious N1 lymph node, which increased the probability of N2/3. The retrospectively smoothed PET data (B+F) are appreciably less sharp than the original images, and N1 SUVmax was lower (9.9),

while the visual score of the N1 lymph nodes remained unchanged (=4). Predicted probability of the GBM with the smoothed PET data was unaffected at 0.2 (still false positive).
